# Control of KirBac3.1 Potassium Channel Gating at the Interface between Cytoplasmic Domains*

**DOI:** 10.1074/jbc.M113.501833

**Published:** 2013-11-20

**Authors:** Lejla Zubcevic, Vassiliy N. Bavro, Joao R. C. Muniz, Matthias R. Schmidt, Shizhen Wang, Rita De Zorzi, Catherine Venien-Bryan, Mark S. P. Sansom, Colin G. Nichols, Stephen J. Tucker

**Affiliations:** From the ‡ Biological Physics Group, Clarendon Laboratory, University of Oxford, Oxford OX1 3PU, United Kingdom,; the § School of Immunity and Infection, University of Birmingham, Birmingham B15 2TT, United Kingdom,; the ¶ Sao Carlos Institute of Physics, University of Sao Paulo, Sao Paulo SP 13560-970, Brazil,; the ‖ Structural Bioinformatics and Computational Biochemistry Unit, Department of Biochemistry, University of Oxford, Oxford OX1 3QU, United Kingdom,; the ** Department of Cell Biology and Physiology and Center for the Investigation of Membrane Excitability Diseases, Washington University School of Medicine, St. Louis, Missouri 63110,; ‡‡ Harvard Medical School, Boston, Massachusetts 02115,; the §§ Institut de Minéralogie et de Physique des Milieux Condensés (IMPMC), CNRS-UMR 7590, Université Pierre et Marie Curie, 75005 Paris, France, and; the ¶¶ OXION Initiative in Ion Channels and Disease, University of Oxford, Oxford OX1 3PT, United Kingdom

**Keywords:** Crystal Structure, Ion Channels, Membrane Proteins, Molecular Dynamics, Potassium Channels, Channel Gating, Kir Channel, KirBac

## Abstract

KirBac channels are prokaryotic homologs of mammalian inwardly rectifying potassium (Kir) channels, and recent structures of KirBac3.1 have provided important insights into the structural basis of gating in Kir channels. In this study, we demonstrate that KirBac3.1 channel activity is strongly pH-dependent, and we used x-ray crystallography to determine the structural changes that arise from an activatory mutation (S205L) located in the cytoplasmic domain (CTD). This mutation stabilizes a novel energetically favorable open conformation in which changes at the intersubunit interface in the CTD also alter the electrostatic potential of the inner cytoplasmic cavity. These results provide a structural explanation for the activatory effect of this mutation and provide a greater insight into the role of the CTD in Kir channel gating.

## Teaser

**Background:** KirBac3.1 is a prokaryotic homolog of eukaryotic Kir channels.

**Results:** A high-resolution crystal structure of a mutant channel reveals a novel open conformation.

**Conclusion:** The intersubunit interface between the cytoplasmic domains controls channel gating.

**Significance:** These findings help define the structures important for gating in prokaryotic and eukaryotic Kir channels.

## Introduction

Recent structural studies of KirBac3.1, an inwardly rectifying potassium (Kir) channel homolog from *Magnetospirillum magnetotacticum*, have provided important insights into the gating mechanism of eukaryotic Kir channels (1, 2). These studies, which involved structures of both closed and open states of the channel, identified global conformational changes associated with opening of the lower helix bundle crossing (HBC)^4^ gate. First, the cytoplasmic domains undergo a rotational movement relative to the plane of the membrane, a conformation termed the “twist state” (1). Second, interactions that link the second transmembrane domain (TM2) and the slide helix with the cytoplasmic domains (CTDs) are crucial for translating the twist conformation into opening the HBC gate (2).

The open state structure was obtained by crystallizing an activatory “gain-of-function” mutant (S129R) (2) that had previously been identified in a random mutagenesis screen (3). However, the S129R mutation was only one of many activatory mutations identified in that study. The mutations clustered in three distinct regions known to be important for the control of Kir channel gating: at TM2 close to bundle crossing, in the CTD, and also near the selectivity filter. The TM2 mutations introduced mainly charged residues and appeared to stabilize the open state of the helix bundle crossing through electrostatic repulsion within the inner pore (2, 3). By contrast, the mechanism by which mutations in the CTD lead to channel activation is more difficult to predict because the role of these domains in channel gating is still poorly understood.

One of the activatory mutations previously identified in the CTD of KirBac3.1 (S205L) is located close to the intersubunit interface between the cytoplasmic domains, and recent studies have implicated an important role for these interfaces in the gating of both prokaryotic and eukaryotic Kir channels (4, 5). Furthermore, a similar activatory mutation (T213I) has also been identified at the equivalent position in KirBac6.1 (3). Thus, a greater understanding of how the S205L mutation affects the structure of KirBac3.1 might help determine the role that the CTD plays in the control of Kir/KirBac channel gating.

In this study, we present the crystal structure of a mutant KirBac3.1 channel at 2.46 Å resolution that shows the effect of the S205L mutation in the context of an open channel, and we have determined the functional and structural consequences of this mutation.

## EXPERIMENTAL PROCEDURES

### 

#### 

##### Protein Expression, Purification, and Crystallization

These were performed as described previously (2). Briefly, following elution from the size exclusion column, the tridecyl-β-maltoside detergent was exchanged to 14 mm HEGA-10 using an Amicon 100-kDa cutoff filtration device, and the protein was concentrated to 6 mg/ml. The channel was crystallized in 10% (v/v) glycerol, 90 mm HEPES (pH 7), 20% (v/v) PEG-400, 5% (w/v) PEG-4000, 2.5% (w/v) PEG-8000, and 10 mm spermidine. Crystals appeared after 2–4 days at room temperature (20 °C) and were harvested using LithoLoops (Molecular Dimensions) and immediately cryo-cooled in liquid N_2_.

##### Data Collection

Data were collected at 100 K using a Pilatus 6M detector at the I-24 beamline of the Diamond Light Source to a resolution of 2.46 Å at a wave length of λ = 0.978 Å. Following the initial space group estimation by Mosflm (6), a full data set was collected from a single crystal.

##### Molecular Replacement and Model Building

Data were indexed and integrated with Mosflm (6), and the space group was confirmed by Pointless (6). Scala was used to scale and merge the integrated data in the space group (6). 5% of the reflections were set aside in the free *R* set. Molecular replacement was carried out with Phaser (7) using a search model derived from KirBac3.1 S129R (Protein Data Bank (PDB) ID 3ZRS), which allowed for instant interpretation of the maps. The model was iteratively refined in real space using Coot (8) and BUSTER 2.10.0 (9), which included a final round of translation, libration, and screw-rotation (TLS) anisotropic refinement, as implemented in BUSTER. The coordinates have been deposited in the PDB with ID 4LP8.

##### KirBac3.1 Complementation and ^86^Rb^+^ Flux Assays

The functional complementation of growth in K^+^ uptake-deficient *Escherichia coli* was performed exactly as described previously (3). Direct measurement of functional activity involved reconstitution of purified protein into liposomes. Briefly, palmitoyloleoylphosphatidylethanolamine and palmitoyloleoylphosphatidylglycerol lipids dissolved in 35 mm CHAPS detergent were mixed at a 3:1 ration to a final concentration of 1 mg/ml. 10 μg of protein was added to 95 μl of lipid mixture and incubated at room temperature for 30 min. Sephadex G-50 columns equilibrated with buffer A (10 mm HEPES (substituted with MES or Tris where appropriate), 450 mm KCl, 4 mm *N*-methyl-d-glucamine, 1 mm EDTA, and 1 mm EGTA, with the buffer pH adjusted to 5.5, 7.5, 8, 8.5, 9, and 9.5 with *N*-methyl-d-glucamine) were partially dehydrated by centrifugation for 10 s at 1000 relative centrifugal force. The mixture of lipids and protein was loaded onto the column and then centrifuged at 700 relative centrifugal force for 10 s to remove the CHAPS detergent. The flow-through fraction containing the newly formed proteoliposomes was collected. To remove external potassium ions, the liposomes were filtered through a partially dehydrated Sephadex column equilibrated with buffer B (10 mm HEPES (substituted with MES or Tris where appropriate), 400 mm sorbitol, 4 mm *N*-methyl-d-glucamine, 1 mm EDTA, and 1 mm EGTA, with the buffer pH adjusted to 5.5, 7.5, 8, 8.5, 9, and 9.5 with *N*-methyl-d-glucamine). To each proteoliposome sample was added 420 μl of buffer B supplemented with ^86^Rb^+^. 60-μl samples were taken at the stated time points. To remove external ^86^Rb^+^, samples were run through a Dowex column. The radioactive content of the proteoliposomes was measured in a scintillation counter, and all values shown represent counts/min.

##### Modeling

All models used for molecular dynamics simulations are based on the KirBac3.1 S129R structure (2) or the KirBac3.1 S129R/S205L double-mutant structure described in this study. Modeler v9.9 (10) was used to add missing residues to the N terminus of the PDB 3ZRS structure before simulation. Two models with wild-type sequence were also generated based on either the coordinates of the 3ZRS (R129S simulation) or 4LP8 (R129S/L205S simulation) structure. To assign protonation states, p*K_a_* values were calculated with PROPKA and PDB2PQR (11) using the PARSE force field (12) at pH 7.

##### Poisson-Boltzmann Calculations

The electrostatic energy profile of K^+^ ions passing through the cytoplasmic pore was calculated using the Adaptive Poisson-Boltzmann Solver (APBS) package (13). Charges and radii were assigned using PDB2PQR, which was also used to add missing atoms of unresolved side chains before calculating electrostatic energies. Energies were calculated using a NaCl bath with an ionic strength of 0.2 m at 298 K. The protein (dielectric constant ϵ = 10) was embedded into a solvent (ϵ = 80). The Cα atom of Arg^129^ in the 3ZRS structure was set to *z* = 0 Å, and the 4LP8 structure was aligned along the selectivity filter of the 3ZRS structure. A step size of Δ*z* = 1 Å was used for APBS calculations.

##### Molecular Dynamics Simulations

All simulations were carried out using the GROMACS 4.5 force field (14). Each model was embedded into a palmitoyloleoylphosphatidylcholine bilayer during a coarse-grained self-assembly simulation (100 ns) using the MARTINI 2.1 force field (15). To neutralize any electrical net charge of the protein, monovalent Cl^−^ and Na^+^ ions were added to the solvent. A Gaussian network model with a 10-Å cutoff and a force constant of 1000 kJ/mol/nm^2^ retained the tertiary and quaternary structures of the channel during coarse-grained simulations. Final coarse-grained snapshots were converted into atomistic systems using a fragment-based approach (16), and two K^+^ atoms were added to the selectivity filter at positions S1 and S3 to simulate a stable conductive state (17). The cavity below the selectivity filter was solvated using VOIDOO (18) to stabilize the pore. Each atomistic system was energy-minimized using at least 5000 iterations of the steepest descent or BFGS quasi-Newtonian minimizer to relax steric clashes. The systems were further minimized during a 1-ns atomistic simulation with positional restraints of 1000 kJ/mol/nm^2^ applied to all heavy atoms of the protein, ensuring equilibration of the solvent. For each equilibrated system, an atomistic simulation of 100-ns duration was performed. Atomistic simulations were carried out at 323 K using the GROMOS96 43a1 force field (19).

## RESULTS

### 

#### 

##### Mutant S205L Directly Affects Channel Activity

**FIGURE 1.**
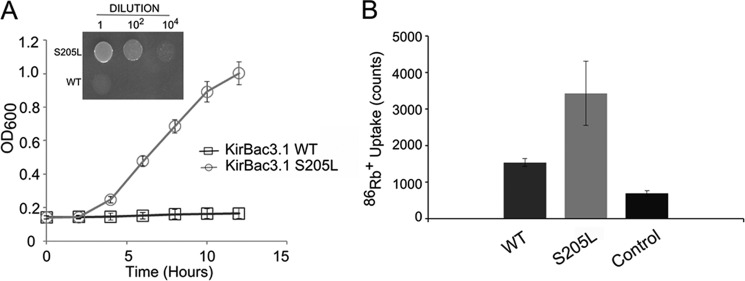
**S205L is an activatory mutation.** *A*, the S205L mutation complements the growth of K^+^-auxotrophic *E. coli* on minimal LB medium containing l mm K^+^ (*inset*). The growth curve shows measurements of culture density in the presence of 1 mm K^+^ over 12 h. *Error bars* (at some points smaller than the symbols) represent S.E. (*n* = 3). *B*, purified S205L protein has a greater functional activity than WT KirBac3.1 in liposomal ^86^Rb^+^ uptake assays (*p* = 0.03). The *bars* represent total rubidium uptake (counts/min) at 30 min at pH 7.5. *Control* represents empty liposomes. *Error bars* represent S.E. (*n* = 3).

The S205L activatory mutation complements the growth of K^+^-auxotrophic *E. coli* on low-potassium media (Fig. 1*A*) (3). However, this assay is not a direct measure of channel activity, so we also examined the activity of the purified KirBac3.1 S205L mutant protein in liposomal rubidium uptake assays.

These assays (Fig. 1*B*) revealed substantially higher activity of the S205L mutant compared with WT KirBac3.1, consistent with a direct effect of the mutation on channel activity. We therefore proceeded to investigate the structural basis for this increase in activity.

##### Crystal Structure of KirBac3.1 with the S205L Mutation

Crystallographic studies of mutants that affect function can provide invaluable insight into the conformational dynamics of a protein. However, both Kir and KirBac channels appear to preferentially crystallize in the closed state, *i.e.* with the HBC gate closed. Indeed, previous structural studies of another activatory KirBac3.1 CTD mutant (Q170A) have shown that it also crystallized in the closed conformation despite having a higher open probability than wild-type KirBac3.1 (1). This suggests that the HBC closed state of the channel represents the lowest energy conformation of the purified protein in detergent micelles and that alternative strategies are required to observe higher energy conformations of the structure such as the open state.

To observe the effects of the S205L mutation in the context of an open conformation channel, we therefore created a double mutation (S129R/S205L) in which the HBC gate was engineered to be open and in which the direct consequences of the S205L mutation might be observed by comparison with our previously reported structure of the KirBac3.1 S129R mutation (2). The mutant channel was therefore crystallized, and the structure was solved to 2.46 Å resolution. The channel crystallized in space group *P*42_1_2, with one molecule in the asymmetric unit. The structure was solved by molecular replacement, and the final model, which was refined to *R*/*R*_free_ = 18.30/24.82% , contains 283 of 301 residues. Of these, 98.6% are in favored regions of the Ramachandran plot with no outliers. Data collection and refinement statistics are shown in Table 1. This structure now represents the most complete and highest resolution structure of a KirBac channel currently available.

**TABLE 1:** **Data collection and structure refinement statistics for KirBac3.1 S129R/S205L (PDB ID 4LP8)**

Space group	*P*42_1_2
Cell dimensions (Å)	105.65, 105.65, 89.90
Cell angles	90.00°, 90.00°, 90.00°
Resolution (highest shell)	24.82-2.46 (2.59-2.46)
*R*_meas_ (highest shell)	0.223 (1.078)
*I*/σ*I* (highest shell)	6.8 (2.0)
Multiplicity (highest shell)	9.0 (9.0)
Completeness for range (highest shell; %)	100.0 (99.99)

**Refinement**	
Resolution (highest shell)	24.82-2.46 (2.59-2.46)
No. of reflections (highest shell)	19,036 (2704)
*R*/*R*_free_ (%)	18.30/24.82
No. of protein atoms	2244
No. of ligand/ion atoms	205
No. of water atoms	187
*B*-factors (Å^2^)	
Protein	49.85
Water	56.56
Inorganic	53.33
Heterogen	61.02
r.m.s.d.*^a^*	
Bond lengths (Å)	0.010
Angles	1.10°

*^a^* r.m.s.d., root mean square deviation.

**FIGURE 2.**
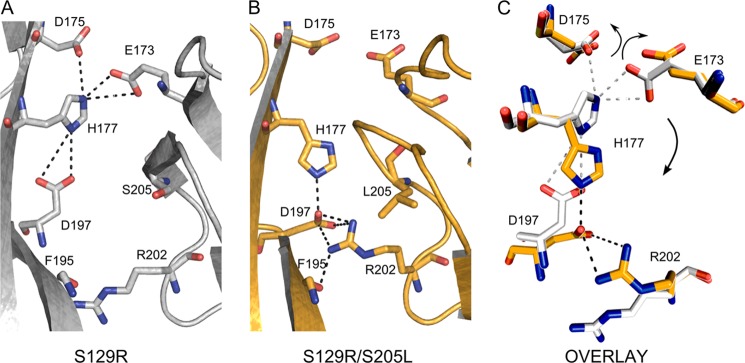
**Structural changes produced by the S205L mutation.** *A*, magnification of the intersubunit connections in the S129R mutant structure. Here, His^177^ engages with Glu^173^ and Asp^175^ to form a triad that may stabilize the tetrameric structure. *B*, magnification of the intersubunit interactions in the S129R/S205L mutant. In this structure, His^177^ has changed its rotamer conformation and interacts with Asp^197^. Also, a novel interaction is observed between Asp^197^ and Arg^202^. His^177^, Asp^197^, and Arg^202^ form a novel triad of interactions at the cytoplasmic domain interface. *C*, alignment of subunit interface residues of the S129R mutant (*gray*) and the S129R/S205L mutant (*gold*). When His^177^ is engaged with Glu^173^ and Asp^175^, these residues are located within the intersubunit space. By contrast, when His^177^ switches its rotamer conformation and interacts with Asp^197^ and Arg^202^, Glu^173^ and Asp^175^ are released from the intersubunit space and line the cytoplasmic pore.

Backbone alignment with the structure of the S129R mutant alone (PDB ID 3ZRS) showed that the two structures are quite similar (root mean square deviation for main chain atoms of ∼0.69 Å). Interestingly, despite this overall similarity, important differences were evident in the vicinity of the S205L mutation. As a consequence of changes in the loop containing the S205L mutation, there is a change in the rotamer conformation of His^177^ that leads to a series of changes in the intersubunit interactions at the cytoplasmic interface (Fig. 2).

**FIGURE 3.**
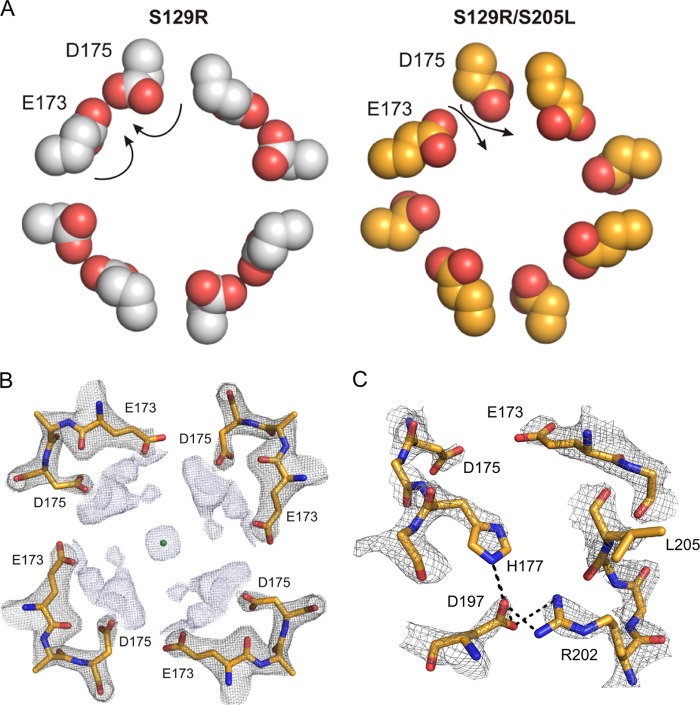
**Mutation S205L results in a ring of negative charges within the cytoplasmic pore.** *A*, top-down view of the arrangement of Glu^173^ and Asp^175^ relative to the cytoplasmic pore in the S129R (*left*, *gray*) and S129R/S205L (*right*, *gold*) mutants. Because of their interaction with His^177^, in the S129R structure, these residues are located within the intersubunit space. However, in the S205L mutant structure, they both point into the cytoplasmic pore. *B*, the cross-section of the 2*F_o_* − *F_c_* map shows a potassium ion (*green sphere*) coordinated by Glu^173^ and Asp^175^. The 2*F_o_* − *F_c_* map is contoured at σ2 for Glu^173^–Glu^175^ (*gray mesh*) and at σ1 for the potassium ion (*light blue mesh*). The potassium ion is probably hydrated, but no water molecules have been modeled around this ion. *C*, side view of the electron density at the cytoplasmic intersubunit interface in the S129R/S205L mutant channel in which His^177^ interacts with Asp^197^, thereby releasing Glu^173^ and Asp^175^ into the inner cavity. The 2*F_o_* − *F_c_* map is contoured at σ2.

In all of the 11 different KirBac3.1 structures available to date (1, 2), the two subunits are connected via the Glu^173^-His^177^-Asp^175^ triad, as shown in Fig. 2*A*. By contrast, in the S205L mutant structure, this interaction is replaced by the novel triad His^177^-Asp^197^-Arg^202^ (Fig. 2*B*). This alternative pairing and charge switching, arising from disruption of the salt bridges centered on His^177^, lead to a notable charge redistribution, with a rotameric switch in the side chains of Glu^173^ and Asp^175^, which now point away from the intersubunit interface and directly into the cytoplasmic pore (Fig. 3). This new conformation therefore provides a possible structural insight into the effect of this mutation and also the role of these intersubunit interactions in channel gating.

**FIGURE 4.**
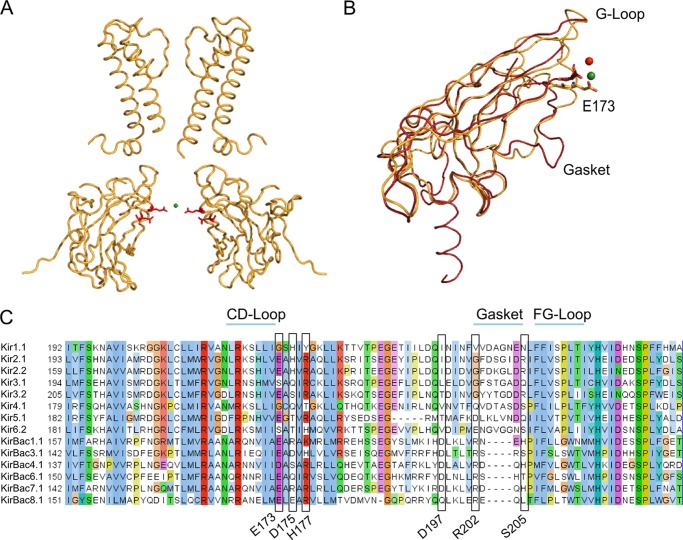
**Ion coordination sites.** *A*, the positions of Glu^173^ and Asp^175^ (*red sticks*) in KirBac3.1. The potassium ion within the cavity (*green sphere*) appears to be coordinated by these residues. For clarity, only two subunits are shown. *B*, structural alignment of the cytoplasmic domains of KirBac3.1 S129R/S205L (*gold*) and Kir3.1 S225E (PDB ID 3K6N; *red*). The Na^+^ ion identified in the Kir3.1 S225E structure is shown as a *red sphere*, whereas the K^+^ ion in KirBac3.1 S129R/S205L is shown as a *green sphere*. Mutation S225E in Kir3.1 mimics the Glu^224/5^ residue found in strongly rectifying Kir2.1 and Kir2.2. This negatively charged residue, along with the gasket loop, provides the cytoplasmic pore with cation coordination sites. They play an important role in facilitating polyamine block and in maintaining normal channel conductance. The gasket loop is not found in prokaryotic Kir channels. *C*, Glu^173^ in KirBac3.1 is highly conserved in both eukaryotic and prokaryotic channels. Other residues relevant to this study are also highlighted.

Additional electron density was identified within the cytoplasmic cavity consistent with the presence of a potassium ion coordinated by Asp^175^ and Glu^173^ (Figs. 3*B* and 4*A*). The distance between the potassium ion and the carboxyl groups is ∼8 Å, indicating that the ion might be either fully or partially hydrated. Despite the higher resolution of this structure, we were not able to resolve water molecules in this part of the channel with any confidence. Interestingly, this potassium coordination site is consistent with similar ion-binding sites in Kir2.2 and Kir3.1 (Fig. 4, *B* and *C*) (20, 21).

##### pH Sensitivity of KirBac3.1

**FIGURE 5.**
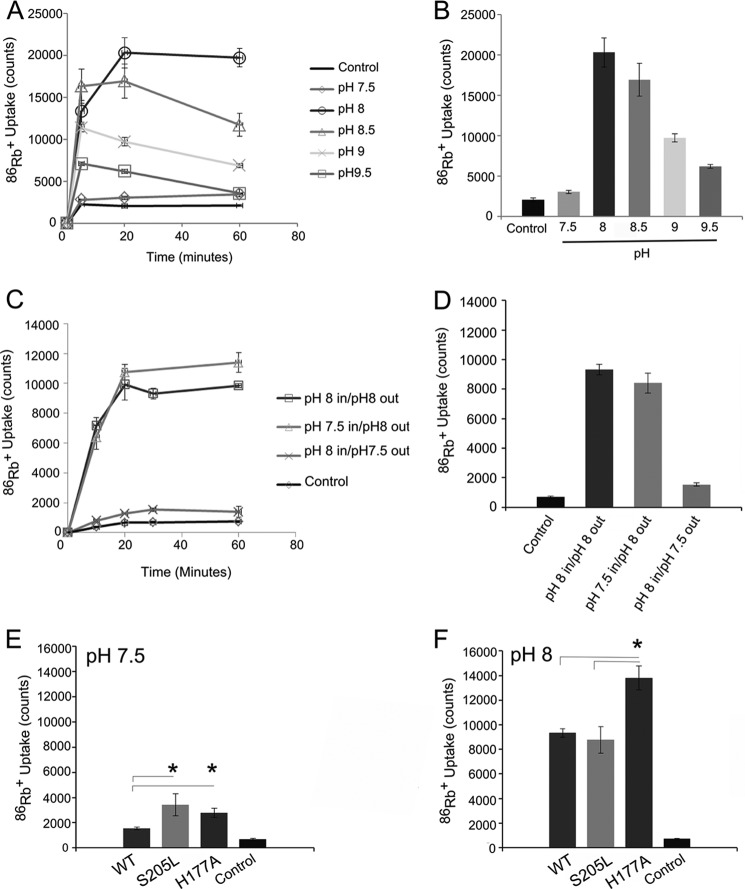
**KirBac3.1 is a pH-sensitive channel.** *A*, time course of ^86^Rb^+^ uptake at different pH values over 60 min. *B*, comparison of ^86^Rb^+^ uptake after 20 min at different pH values. Data are presented as the mean ± S.E. (*n* = 3). Channel activity reached a maximum at pH 8.0 and steadily decreased as the pH became more alkaline. *C* and *D*, the pH sensitivity is one-sided. Full activity of the channel could be obtained by changing only the extraliposomal pH to 8, whereas adjusting the internal pH to 8 resulted in low channel activity. This indicates that the majority of the channels are oriented uniformly in the liposomes and that the pH sensitivity originates from the side of the channel facing the external solution. The data are presented as the mean ± S.E. (*n* = 3) for all time points. *E* and *F*, steady-state ^86^Rb^+^ uptake of WT KirBac3.1 compared with S205L and H177A at pH 7.5. Both S205L and H177A increased the activity of the channel, but neither abolished the pH sensitivity of the channel. Mutation S205L appeared to be functionally silent at pH 8 compared with the WT channel. Mutation H177A remained more active than both the wild-type channel and the S205L mutant. *Error bars* represent S.E. (*n* = 3). *, *p* < 0.05.

The structural changes observed at the cytoplasmic interface involve His^177^, suggesting that pH might influence this residue. We investigated the possibility that KirBac3.1 activity might be pH-dependent using ^86^Rb^+^ flux assays. Wild-type KirBac3.1 exhibited very low functional activity in the pH 5.5–7.5 range, but there was an almost 10-fold increase in activity at pH 8.0, which steadily declined as the pH was raised to more alkaline values (pH 8.5–9.5) (Fig. 5, *A* and *B*). Importantly, channel activity responded only to pH changes in the extraliposomal buffer (Fig. 5, *C* and *D*). This indicates not only a specific effect of pH on one side of the channel but also that the channel protein does not randomly insert into the liposomal membranes during reconstitution, an observation that has also been confirmed for KirBac1.1 (22). However, the absence of known blockers and/or ligands that act asymmetrically means that it is difficult to predict whether this extraliposomal side corresponds to the cytoplasmic or extracellular side of the protein.

We next investigated whether mutations at the cytoplasmic interface influence this pH sensitivity. The S205L mutation did not abolish the pH dependence of the channel, but it is interesting to note that even though S205L was more active than the WT channel at pH 7.5, it was less sensitive to alkaline activation, so at pH 8, the S205L activity was the same as that of the WT channel (Fig. 5, *E* and *F*). We also examined the effect of mutating His^177^, which plays a key role in defining these intersubunit interactions. The H177A mutant was similarly more active than the WT channel at pH 7.5, but it also showed a significant increase in activity from pH 7.5 to 8 (Fig. 5, *E* and *F*), indicating that His^177^ is unlikely to act as the main pH sensor in KirBac3.1. In structural terms, the H177A mutation may be expected to mimic some aspects of the S205L mutation because it would also prevent formation of the Glu^173^-His^177^-Asp^175^ triad, thereby releasing both Glu^173^ and Asp^175^ to line the cytoplasmic vestibule. This is consistent with the observed activatory phenotype of the H177A mutant.

##### Mechanism of Activation by S205L

**FIGURE 6.**
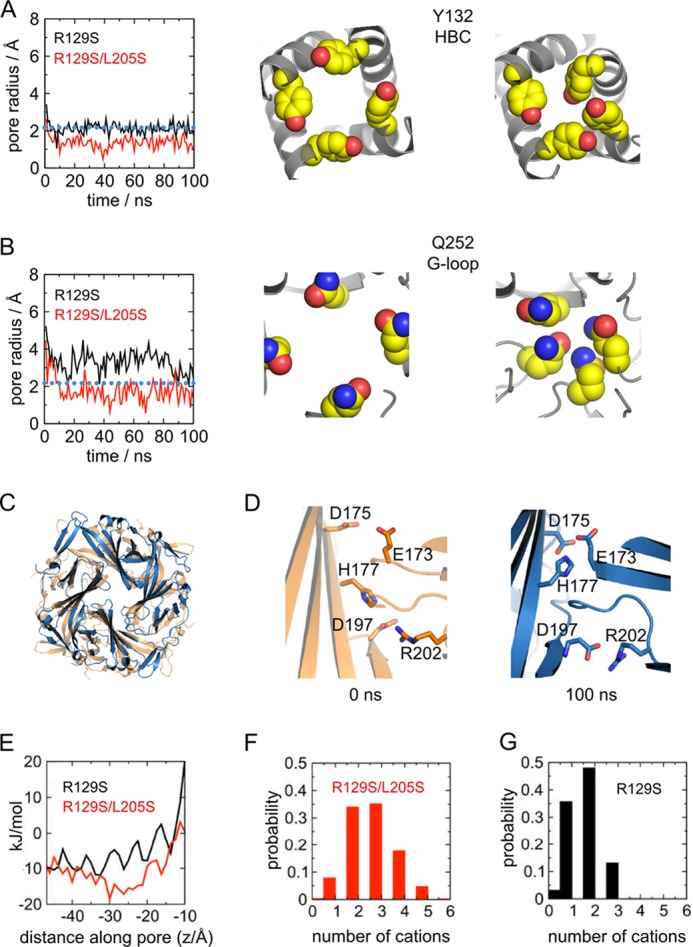
**Effect of S205L on stability of the open state conformation.** *A*, molecular dynamics simulations of the single-mutant (R129S) and double-mutant (R129S/L205S) structures, *i.e.* where residues in the mutant structure were converted back to wild-type serine. *Left*, the pore radius, calculated by HOLE (40) along the pore axis, is shown for the HBC at Tyr^132^. The radius of a hydrated K^+^ ion is indicated by the *dotted blue line*. For times *t* = 0 ns and *t* = 100 ns, snapshots of the respective locations obtained in the R129S/L205S simulation are shown (*right*). *B*, as shown for *A*, but the radius was calculated at the position of the G-loop (Gln^252^). Although the G-loop closes only in the R129S/L205S simulation, the side chains of the HBC close the pore in both simulations. *C*, overlay of the CTD of the S129R/S205L mutant structure with a snapshot at *t* = 100 ns of the R129S/L205S simulation. No significant rotation of the CTD is observed. *D*, comparison of the intersubunit interface in the R129S/L205S simulation. At 0 ns, the side chains of Glu^173^ and Asp^175^ point toward the pore, and His^177^ points down, interacting with Asp^197^ and Arg^202^. At 100 ns, His^177^ has adopted the closed state conformation, and the side chains of Glu^173^ and Asp^175^ turn into the outward-facing position, also adopting the closed state orientation. *E*, APBS profiles of S129R and S129R/S205L. The Poisson-Boltzmann potential energy of a K^+^ ion, calculated along the pore axis of the CTD, is markedly lower in S129R/S205L due to reorientation of Glu^173^ and Asp^175^, rendering passage of K^+^ through the CTD pore more energetically favorable. *F*, number of cations (Na^+^) in the cytoplasmic pore (−16 Å ≤ *z* ≤ −37 Å) during the R129S/L205S simulation. *G*, number of cations (Na^+^) in the cytoplasmic pore during the R129S simulation.

To probe the mechanism of activation, we undertook a series of computational approaches (Fig. 6). We examined the effect of these negatively charged residues on the electrostatic potential of the cytoplasmic pore by comparing the APBS calculations of the S129R mutant and the S129R/S205L double mutant. This analysis revealed a significant decrease in the energetic barrier to potassium permeation in the cytoplasmic pore at the level of Glu^173^ and Asp^175^ compared with the KirBac3.1 S129R structure (Fig. 6*E*).

Interestingly, an alignment of KirBac3.1 with eukaryotic Kir channels showed that Glu^173^ is conserved in all KirBac channels and the strongly rectifying Kir channels and corresponds to Glu^225^ in Kir2.2 (Fig. 4*C*). This glutamate was originally identified as a coordination site for polyamines in the cytoplasmic vestibule (21, 23–27) and is therefore an important factor in the kinetics of inward rectification. It has also been found to influence unitary conductance in both Kir2.1 and Kir2.2 (23, 25, 27, 28).

The significant change in the electrostatic potential produced by the S205L mutation will result in a more attractive environment for cations, and an increased ability to concentrate permeant cations in the CTD might facilitate ion conductance through the channel. Structural changes at this intersubunit interface may also affect channel gating, but our results show that they clearly change the electrostatic potential within the cytoplasmic pore.

##### Changes in the Energetic Stability of the CTD

To further examine the consequences of the S205L mutation on the CTD, we also performed a series of atomistic molecular dynamics simulation. During a 100-ns simulation of the S129R mutant (PDB ID 3ZRS), the structure appeared to remain stable, and no further opening (or closing) was observed, indicating a high degree of stability (data not shown). We therefore ran 100-ns-long simulations of the S129R and S129R/S205L open state structures with the mutant side chains changed back to the wild-type side chain, *i.e.* PDB ID 3ZRS (R129S) and ID 4LP8 (R129S/L205S). This would allow any constraints keeping these mutant structures open to be released and enable observation of any potential relaxation back to the closed conformation.

Intriguingly, during these simulations, major conformational changes were observed. In all simulations, there is a narrowing at the HBC gate due to reorientation of TM2 at the Tyr^132^ side chain (Fig. 6*A*). Narrowing of the pore similar to that seen in the S129R mutant (2) is also observed at the upper constriction in TM2 (Leu^124^). However, a major difference is observed at the G-loop located just below the HBC gate, which has been proposed to act as an additional gating element within the CTD (29, 30). Closure of the G-loop, which reduced the radius of the conduction pathway from 5 to <2.2 Å, *i.e.* well below that of a hydrated K^+^ ion, is observed during simulation of the double-mutant structure (R129S/L205S), but not of the single-mutant structure (R129S) (Fig. 6*B*). These results suggest that the putative G-loop gate has a greater propensity to close in the double-mutant structure and that this new structure may therefore be representative of a higher energy open conformation.

We also examined whether any changes occur at the CTD interface during the simulation. No change in the twist of the CTD relative to the transmembrane domain is observed in the double mutant (Fig. 6*C*), but the interface interactions do undergo a significant change (Fig. 6*D*). The His^177^ side chain initially interacts with Asp^197^ and Arg^202^ (*t* = 0 ns), but in three of the four subunit interfaces, subtle twists occur in the Gβ and Hβ strands, allowing His^177^ to interact with Glu^173^ and Asp^175^ at the end of the simulation (*t* = 100 ns). This reverts these negatively charged side chains to their original conformations observed in the S129R structure (PDB ID 3ZRS) and highlights an important role for His^177^ at this interface.

## DISCUSSION

The results presented here show that the S205L activatory mutation in the CTD stabilizes KirBac3.1 in a novel high-energy open conformation and highlight an important role for the CTD intersubunit interfaces in the control of channel activity. In all of the available KirBac3.1 structures to date, both the Glu^173^ and Asp^175^ residues appear to play a role in maintaining the tetrameric assembly of the CTD. However, in the structure presented here, the S205L mutation disrupts the coordination of these residues, with His^177^ thereby changing the electrostatic potential of the cytoplasmic pore. Consistent with the reorientation of these side chains, we also observed more cations in the cytoplasmic pore over the time course of the double-mutant simulation than in the single-mutant simulations (Fig. 6, *F* and *G*). Glu^173^ is a highly conserved residue within the CTD of both prokaryotic and eukaryotic Kir channels (Fig. 4*C*) and has been identified as a K^+^ ion coordination site in both Kir2.2 (20) and Kir3.1 S225E mutant channel structures (21). It has been proposed that the negative charges in the cytoplasmic pore act to increase the local concentration of K^+^ and facilitate transfer of ions to the transmembrane region. Indeed, removal of the negative charge at position 224 in Kir2.1 reduces the unitary conductance of the channel (23), whereas other studies have shown that a negative charge in this position is necessary for maximal K^+^ conduction (28, 31). Interestingly, His^177^ in KirBac3.1 is equivalent to His^216^ in Kir6.2, which has been identified as the principal determinant of the pH-dependent polyamine block observed in Kir6.2 (32).

Changes in the intersubunit connections at the CTD interface are also likely to be important for the control of channel gating, and recent FRET studies of KirBac1.1 have established that these interfaces do indeed undergo significant structural changes during gating (4). It is also important to note that activatory mutations at this interface have been found in other KirBac channels (3). Thus, although our results demonstrate that changes in the electrostatic properties of the CTD pore are likely to contribute to the activatory effect of the S205L mutation, we cannot exclude direct effects of the S205L mutation on the kinetics of channel gating.

We also now report that KirBac3.1 is a pH-sensitive channel. A degree of pH sensitivity is an intrinsic feature of all Kir channels and is thought to be facilitated by a number of titratable inter- and intrasubunit interactions. The differences in pH sensitivity among members of the Kir channel family seem to arise from connections that stabilize either the closed or open state (33–37). KirBac1.1 has also previously been shown to be inhibited by acidic pH (38), but KirBac3.1 is the only prokaryotic Kir channel that exhibits regulation within a more neutral pH range. Further studies will be required to determine the asymmetry of this response, but KirBac3.1 may now prove useful as a model system to further probe the overall pH-gating mechanism observed in eukaryotic Kir channels.

A recent structure of the Kir3.2-βγ complex showed that the CTD undergoes only a modest (4°) twist relative to the transmembrane domain upon βγ binding, and it has been proposed that this may lower the energetic barrier between the open and closed states (5). In our original structure of the open state S129R mutant, we showed that a 23° twist of CTD facilitates a network of interactions between the transmembrane domain and CTD, helping to stabilize the open state of the channel. However, in our molecular dynamics simulations, we observed no “untwisting” of the CTD during closure of the double-mutant structure. This is possibly because the relatively large energetic barrier for rotation of the CTD is not overcome during the limited time scale of our simulation or because the twisting observed in KirBac3.1 may be different from the more limited twisting observed in eukaryotic Kir channels. The C-linker region that connects the transmembrane domain to the CTD in the prokaryotic channel is markedly different from that in eukaryotic channels and may also account for this difference.

Nevertheless, although the structural consequences of the S205L mutation appear to be quite subtle, our molecular dynamics simulations suggest that even such subtle changes induce a novel higher energy conformation that might lower the energetic barrier between the closed and open states of the channel and therefore explain the activatory effect of this mutation. An almost identical activatory mutation has been observed in KirBac6.1 (3), and although this region may be different in eukaryotic Kir channels due to the presence of the adjacent gasket (Fig. 4), a similar mutation was also identified in eukaryotic Kir channels (36). Therefore, the structural mechanisms identified here may be of relevance to both prokaryotic and eukaryotic Kir channel gating.

In other ion channels, the ability to observe structural changes induced by functional mutations has often proven elusive (39), perhaps because when isolated from the membrane, they prefer to crystallize in their lowest energy conformation despite the profound functional effects induced by these mutations. Indeed, Kir/KirBac channels preferentially crystallize in the closed conformation. However, the approach taken here of stabilizing the HBC in an open conformation with the S129R mutation appears to have overcome this problem and has allowed crystallization of the S205L mutant in the context of an open state channel. Similar approaches to crystallographic studies of other ion channel mutations may therefore enable further expansion of the conformational landscape for these dynamic proteins.

## Footnotes

4 The abbreviations used are:

HBC: helix bundle crossing
TM2: second transmembrane domain
CTD: cytoplasmic domain
PDB: Protein Data Bank.
